# Statistical optimization of experimental parameters for extracellular synthesis of zinc oxide nanoparticles by a novel haloalaliphilic *Alkalibacillus* sp.W7

**DOI:** 10.1038/s41598-021-90408-y

**Published:** 2021-05-25

**Authors:** Hend M. H. Al-Kordy, Soraya A. Sabry, Mona E. M. Mabrouk

**Affiliations:** 1 Botany and Microbiology Department, Faculty of Science, Damanhour University, Damanhour, Egypt; 2 Botany and Microbiology Department, Faculty of Science, Alexandria University, Alexandria, Egypt

**Keywords:** Biotechnology, Microbiology

## Abstract

Green synthesis of zinc oxide nanoparticles (ZnO NPs) through simple, rapid, eco-friendly and an economical method with a new haloalkaliphilic bacterial strain (*Alkalibacillus* sp. W7) was investigated. Response surface methodology (RSM) based on Box-Behnken design (BP) was used to optimize the process parameters (ZnSO_4_.7H_2_O concentration, temperature, and pH) affecting the size of *Alkalibacillus*-ZnO NPs (Alk-ZnO NPs). The synthesized nanoparticles were characterized using UV–visible spectrum, X-ray diffraction (XRD), Scanning electron microscope-energy dispersive X-ray spectroscopy (SEM–EDX), Transmission electron microscopy (TEM), Fourier transform infrared spectroscopy (FTIR) and Zeta potential. The UV–Vis spectrum of ZnO NPs revealed a characteristic surface plasmon resonance (SPR) peak at 310 nm. XRD pattern confirmed the hexagonal wurtzite structure of highly pure with a crystallite size 19.5 nm. TEM proved the quasi-spherical shape nanoparticles of size ranging from 1 to 30 nm. SEM–EDX showed spherical shaped and displayed a maximum elemental distribution of zinc and oxygen. FTIR provided an evidence that the biofunctional groups of metabolites in *Alkalibacillus* sp.W7 supernatant acted as viable reducing, capping and stabilizing agents.

## Introduction

Nanoparticles (NPs) with at least one dimension sized from 1 to 100 nm^1^exhibit unique features owing to their extremely small size and high surface area to volume ratio, which have attributed to the significant differences in the properties over their bulk counterparts^1,2^. The increasing research in the field of nanotechnology has influenced many areas such as energy, medicine, electronics, environment, catalysts and space industries^3–5^.

The synthesis of nanoparticles with the help of microorganisms has no harmful impacts, does not require use of hazardous chemicals, energy efficient, simple, clean, economical, and provides greater biocompatibility especially in the clinical and biomedical applications^6^. In addition the various natural materials in microorganisms function as a potential biofactory for the reduction and stabilization of the nanoparticles^7,8^. Moreover, simple downstream processing provides greater biocompatibility in the uses of nanoparticles for pharmaceutical and biomedical applications and effective sources for high productivity and purity^9,10^. One of the few disadvantages for microbial synthesis of nanoparticles is the tedious purification steps and poor understanding of the mechanisms controlling the shape, size, and mono dispersity.Also, scaling up the production processing for industrial applications is a challenge for its commercialization. Another challenge is slection of biocatalyst and optimization of reaction condition to achieve better yield and small size^9,10^.

Zinc oxide nanoparticles (ZnO NPs) are one of the most important metal oxide with many significant features such as chemical and physical stability, high catalytic activity, effective antibacterial activity as well as intensive ultraviolet (UV) and infrared (IR) adsorption^11^. They are used in an array of products and wide range of applications^1,12,13^.

Traditionally, ZnO NPs synthesized by various physicochemical methods suffer from drawbacks such as high cost, requirement of elevated temperature, high pressure, specialized equipment, use of toxic and environmentally hazardous chemicals; resulting in high energy consumption and the generation of large amounts of waste which pose environmental risks^1^. Microbes such as bacteria, fungi, and yeast play an important role in the biological synthesis of metal and metal oxide NPs. Nevertheless, the biological synthesis of ZnO NPs using microbes still remains unexplored^1^.

The formation of nanomaterials by extremophiles is a promising approach for the biosynthesis of metallic nanoparticles. Extremophiles are important in the synthesis of NPs because they allow for the adjustment of the environment to fine-tune size, shape, stability, and capping proteins. This in turn optimizes the NPs for the large number of applications that they may fulfill^14^. From the literature survey, bacteria isolated from a challenged environment like salt alkaline habitats have never been explored as potential biofactories for the synthesis and stabilization of ZnO NPs. In an early report, *Halomonas elongata* IBRC-M 10214 has been recognized as a bio-factory, capable of producing ZnO NPs^15^, whereas there was no literature available on nanoparticles synthesis by genus *Alkalibacillus.*

Synthesis methods that control the size and shape of nanoparticles are useful in boosting their chemico-physical properties. It is imperative to optimize the parameters employed in the synthesis protocol to achieve good monodispersity, greater particle stability and excellent biocompatibility^16,17^. The strong relationship between size and properties of nanoparticles has provided numerous chances for many scientific areas^18^.

Optimization of factors such as temperature, pH value, precursor concentration, mixture ratio, and reaction time, and their interaction affect the biological synthesis of metal nanoparticles^19^ have been very poorly evaluated for ZnO nanoparticles^20^. Statistical experiment strategy such as response surface methodology (RSM) is used to determine the relationship between the response variable (e.g., NPs size) and the factors affecting their size. Further, it determines the accurate optimum value (s) of the examined variables that give (s) a maximum or minimum optimal response, depending on the product or on the process in question^16^. This method consists of a number of mathematical and statistical techniques which improves and optimizes processes through evaluating the relationship between the independent variables and responses^21^. This method was successfully applied in many areas of biotechnology, including some recent studies for green synthesis of nanoparticles^16,17,22,23^.

Therefore, this innovative study explores an efficient, single step, green, environment-friendly and low-cost method for extracellular biosynthesis of ZnO nanoparticles using *Alkalibacillus* sp.W7 as a reducing as well as surface stabilizing agent. The reaction parameters including temperature, concentration of Zinc sulphate, and pH value that affect the average diameter size of ZnO nanoparticles were statistically optimized with the Box–Behnken experimental design method. The biosynthesized ZnO NPs were characterized and confirmed through various techniques such as UV–visible spectroscopy followed by SEM, EDX, TEM, XRD, and FTIR.

## Results and discussion

### Extracellular synthesis of Alk-ZnO NPs

**Figure 1:**
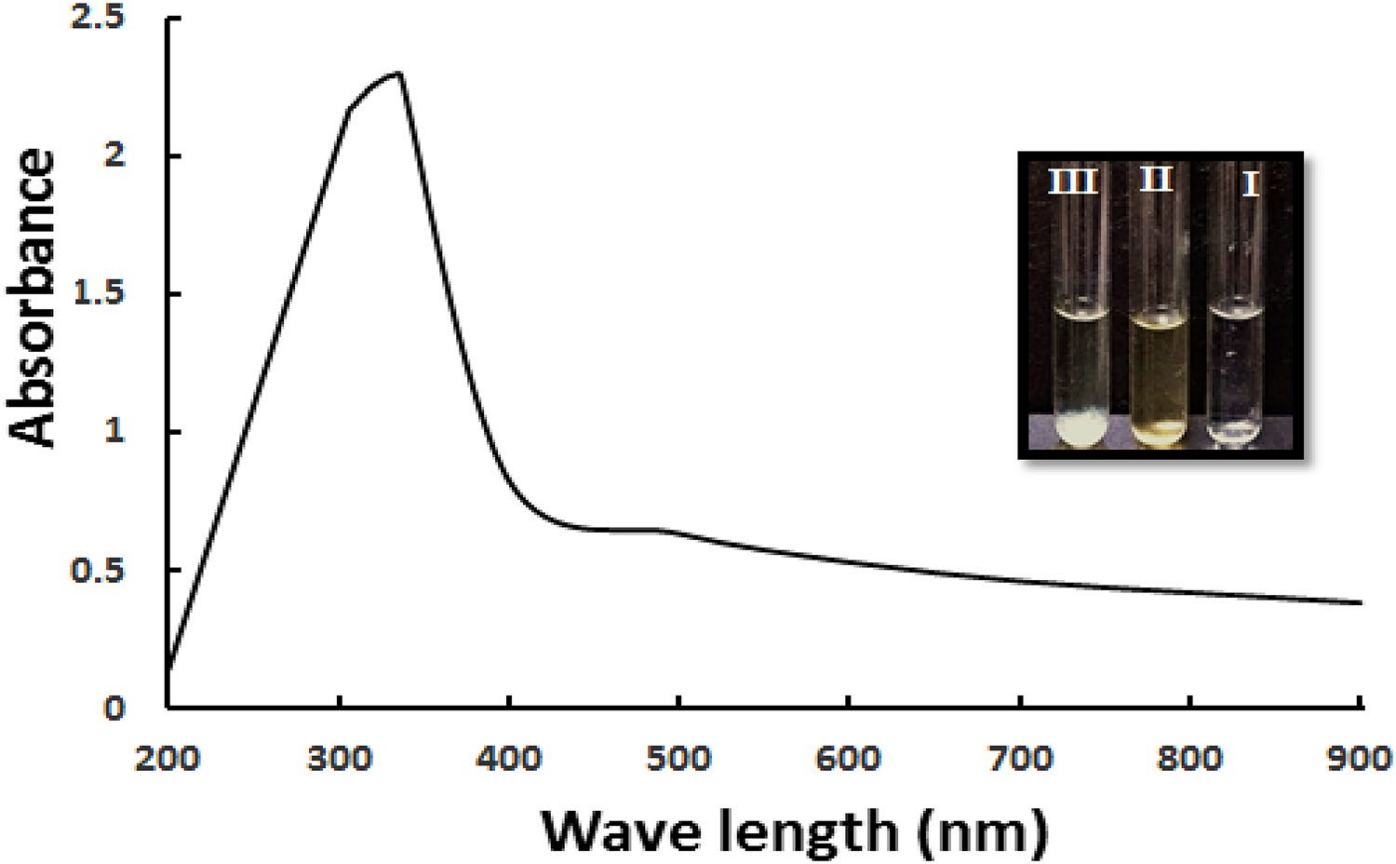
UV–vis absorption spectra of biosynthesised Alk-ZnO NPs, with maximum absorption at 334 nm. Inset shows visual observation of color change (I) Pure precursor (ZnSO_4_.7H_2_O), (II) reducing agent (culture supernatant), (III) white clusters of Alk-ZnO NPs formed after mixing reducing agent and precursor.

To the best of our knowledge, this is the first report employing *Alkalibacillus* sp. W7 as a natural nanofactory for extracellular synthesis of ZnO NPs. The preliminary detection of nanoparticles formation was distinguished through visual observation of cloudy white clusters deposited at the bottom of the tube compared to control (Fig. 1). The cloudy white precipitate arised as a result of reduction of Zn^+2^ ions to elemental Zn^0^ by active metabolites in the culture filtrate and is due to the excitation of surface plasmon resonance (SPR) of the ZnO nanoparticles^24^. It has been demonstrated that the proteins, enzymes as well as other active metabolites secreted by microorganisms are the principal biomolecules involved in the formation of metal/metal oxide nanoparticles^4,25^. It was found that the UV–vis absorption spectrum of biosynthesized Alk-ZnO NPs demonstrated SPRcharacteristic maximum absorption peak at 334 nm (Fig. 1), several biofabricated ZnO NPs revealed comparable absorption peaks^20,24,26^. No other peaks were observed in the spectrum, indicating high purity and crystallinity of Alk-ZnO NPs. The absorption peak depends on size and shape of synthesized NPs.

### Optimization parameters of Alk-ZnO NPs size through Box Behnken design

The average diameter of metal nanoparticles can be affected by some factors including pH, salt concentration and temperature of incubation^21^. Response surface methodology was applied to investigate the relation between the factors (pH, temperature and ZnSO_4_.7H_2_O concentration) on the average size of Alk-ZnO NPs. For each trial, the average particle size acquired from the regression equation for the 15 combinations are illustrated in Table 1.

**Table 1:** Design matrix of Box–Behnken experiments showing coded and actual values of the evaluated independent variables along with response.

Trial	Variable coded and actual values			Average size (nm) of ZnO NPs
	X_1_ pH	X_2_ Temperature (°C)	X_3_ ZnSO_4_.7H_2_O (mM))	
1	1(9)	0 (30)	1(10)	24.03
2	0 (8)	0 (30)	0 (8)	21.4
3	1(9)	0 (30)	− 1(2)	36.46
4	0 (8)	0 (30)	0 (8)	20.5
5	1(9)	1(50)	0 (8)	27.6
6	− 1(7)	1(50)	0 (8)	32.7
7	− 1(7)	0 (30)	1(10)	26.4
8	0 (8)	1(50)	1(10)	27.14
9	0 (8)	1(50)	− 1(2)	33.6
10	− 1(7)	0 (30)	− 1(2)	28.02
11	− 1(7)	− 1(20)	0 (8)	29.4
12	0 (8)	0 (30)	0 (8)	20.7
13	0 (8)	− 1(20)	1(10)	26.02
14	1(9)	− 1(20)	0 (8)	25.5
15	0 (8)	− 1(20)	− 1(2)	31.5

It was observed that the smallest sized particles were produced from trials 2, 4, and 12. The determination coefficient (R^2^) values provide a measure of how much variability in the observed response can be explained by the experimental factors and their interactions. The closer the R^2^ value to 1, the stronger the model is, the better it predicts the response and has a very high correlation^22,27^. The determination coefficient (R^2^) of the model (0.9654) indicates 96.54% of variability in the response. Therefore, the present R^2^-value indicates a very good fit between the observed and predicted responses and implies that the model is reliable in the present study.

Analysis of variance (ANOVA) was used to evaluate the significance and adequacy of the regression model. According to ANOVA results (Table 2), the model was highly appropriate; the relationship between the independent variables and the average size of nanoparticles was evident from low *P*-value (0.00378).

**Table 2:** Analysis of variance (ANOVA) of the fitted quadratic polynomial model.

Source of variations	Degree of freedom	Sum of squares	Mean square	Fishers’s function F-value	Level of significance (*P*-value)	Determination coefficient R^2^
Regression	9	305.371	33.93	15.533	0.003783	0.96546
Residual	5	10.922	2.184			
Total	14	316.293				

The value of adjusted-R^2^ (0.9033) was also very high indicating a high significance of the model^22^ and suggested that the total variation of 90.33% average diameter size of nanoparticles is attributed to the independent variables and only about 9.67% of the total variation cannot be explained by the model.

All values of model coefficients were calculated by multiple regression analysis. The significance of each coefficient was determined by the Student’s *t*-test and *P*-value, as illustrated in Table 3. Based on the analysis of regression coefficients of the quadratic model, among the three tested variables, the linear effects of reaction pH (X_1_), and reaction temperature (X_2_) exhibited a negative relationship on particle size, while zinc sulphate concentration (X_3_) exhibited a positive relationship.

**Table 3:** Regression analysis for optimizing ZnO nanoparticles size using Box–Behnken design.

Variables	Coefficients	Main effect	*t* Stat	*P* value	Confidence level (%)
Intercept	230.9812	461.962	4.441171	0.00000	100
X_1_ (pH)	− 50.2081	− 100.416	− 4.0173	0.010148	99.10991
X_2_ (°C)	− 1.00097	− 2.00194	− 2.06055	0.094367	91.39283
X_3_ (ZnSO_4_)	2.895308	5.790616	1.762809	0.138222	85.95297
X_1_X_2_	− 0.0418	− 0.08359	− 0.86907	0.424555	58.14298
X_1_X_3_	− 0.79829	− 1.59659	− 4.56962	0.006004	99.43325
X_2_X_3_	− 0.00029	− 0.00059	− 0.02589	0.980343	1.992987
X_1_X_1_	3.549259	7.098519	4.601892	0.00583	99.48717
X_2_X_2_	0.020132	0.040264	5.105393	0.003753	99.66835
X_3_X_3_	0.22403	0.448059	3.309396	0.021255	98.13707

*P*-value indicates the significance of each of the coefficients which is important to understand the pattern of the mutual interaction among variables. The smaller the magnitude of the *P*, the more significant is the corresponding coefficient. According to the degree of significance (*P* ≤ 0.01, significant at 99% level), the linear term of pH (X_1_) had the most dramatic negative significant effect on NPs size among all linear effects. In contrast, the quadratic terms for pH (X_1_^2^), temperature (X_2_^2^) and metal concentration (X_3_^2^) had a significant positive effect; meaning that they could be considered as limiting factors and little variation in their values will alter the size of nanoparticles. In addition, only interaction between pH (X_1_) and metal concentration (X_3_) was noted to have a negative significant effect.

**Figure 2:**
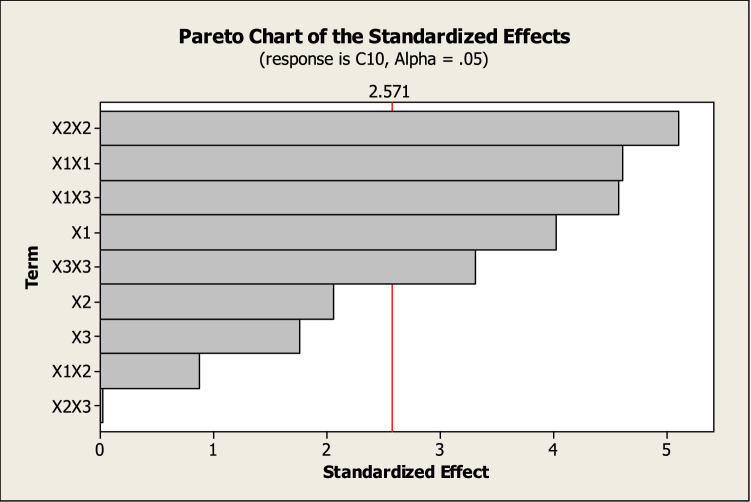
A Pareto chart illustrating the main effects of Box-Behnken result, with the order of significance of the variables affecting Alk-ZnO NPs size. Bars that exceed the vertical line indicate the significance of the terms (*P* < 0.05).

The Pareto chart identified as a useful tool for determining the most significant effects^28^ was used to illustrate the order of significance of the different factors on Alk-ZnO NPs size. Each bar length on a standardized Pareto chart (Fig. 2) is proportional to the absolute value of its related estimated effect or regression coefficient.

Design Expert software was used to fit a second order polynomial model to the experimental results to predict the size of NPs within the experimental constraints. The following second order polynomial equation represents the empirical relationship between NP size and the three independent variables:

$${\mathbf{Y}}_{{\left( {{\mathbf{NPsSize}}} \right)}} = 230.9812 - 50.2081{\mathbf{X}}_{1} - 1.00097{\text{X}}_{2} + 2.895308{\mathbf{X}}_{3} - 0.0418{\mathbf{X}}_{1} {\mathbf{X}}_{2} - 0.79829{\mathbf{X}}_{1} {\mathbf{X}}_{3} - 0.00029{\mathbf{X}}_{2} {\mathbf{X}}_{3} + 3.549259 {\mathbf{X}}_{1}^{2} + 0.020132{\mathbf{ X}}_{2}^{2} + 0.22403{\mathbf{X}}_{3}^{2}$$

The coefficients sign determines the response performance. Here, coefficient with positive effect means decrease in particle size, while a negative effect means increase in particle size. In addition, at higher absolute value of a coefficient, the effect of variable is higher. Therefore, according to the previous equation, pH of the reaction is the variable with the strongest influence on NPs size.

**Figure 3:**
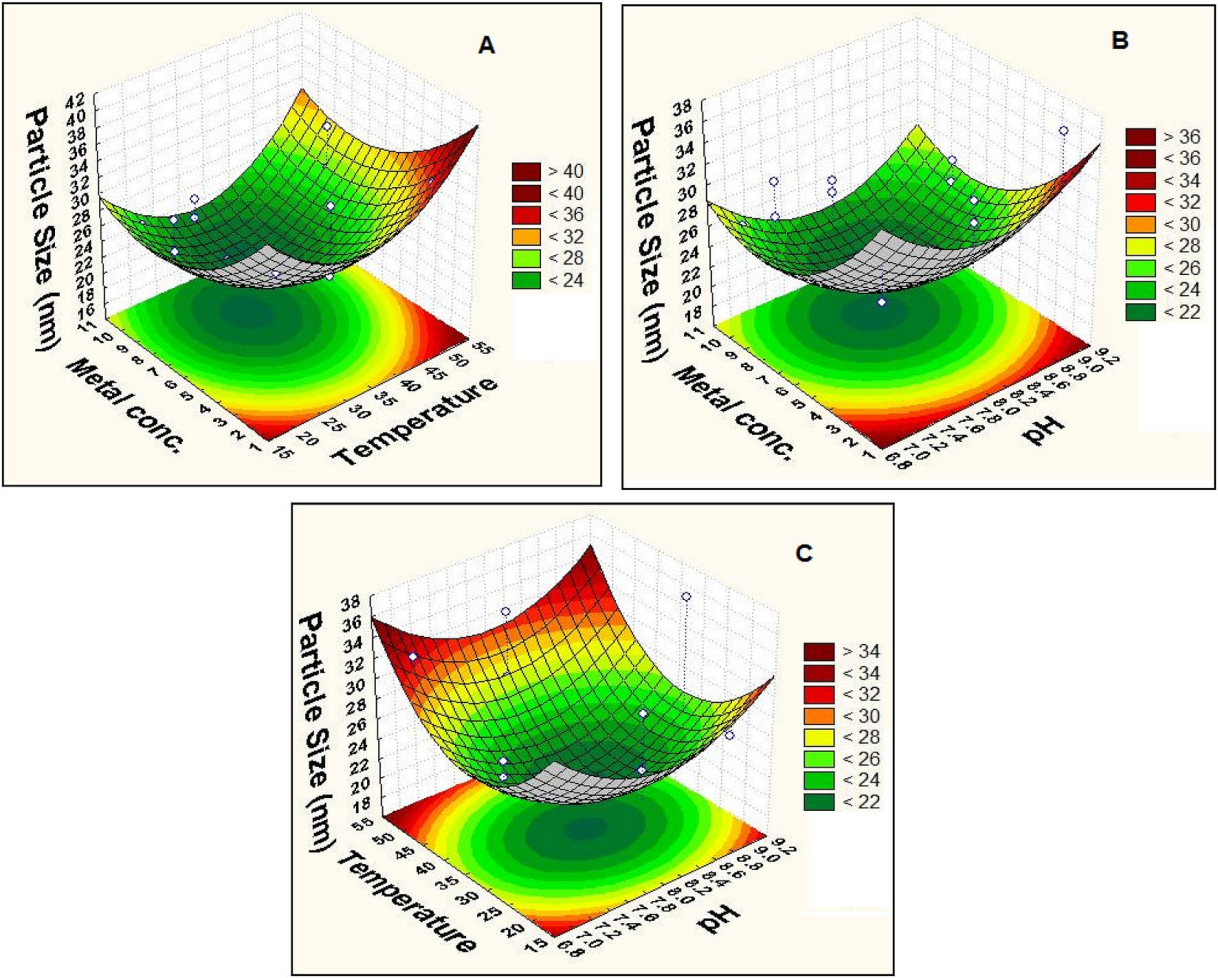
Three-dimensional response surface graphs and contour plots showing the effect of independent variables on the average size of Alk-ZnO NPs; (**A**) Effect of metal concentation and temperature, (**B**) Effect of metal concentation and pH and (**C**) Effect of temperature and pH.

From polynomial models, response surface curves were plotted using Design-Expert 7.0, a trial version software to determine the effects of factors and their combinations that optimize the response. These three-dimensional plots and their respective contour plots provided a visual interpretation of the interaction of two test variables with the third one held at a constant level and facilitated the location of optimum values of the variables for synthesis of the smallest NPs. Significance of the interaction plots between the corresponding variables was indicated by an elliptical or circular or saddle nature of the contour plots^23^. The contour plots (Fig. 3) reveal that the optimal point for decreasing the average diameter of Alk-ZnO NPs was located inside the experimental region, which confirms the adequacy of the independent variable concentration ranges used in this study. The response surfaces obtained were concave, indicating that the operating conditions were well-defined and optimum. Figure 3A represents the interaction between temperature and metal concentration and reveals that the average diameter size decreased with gradual increasing in both metal concentration and temperature. This might be explained that increasing metal ion concentration to a certain point allows particle growth at a faster rate and generates NPs with smaller size. Moreover, higher metal precursor concentration in the reaction mixture may increase the aggregation of the growing NPs, which resulted in the formation of larger NPs size^21,29^. Phanjom &Ahmed^30^ reported that AgNO_3_ concentration up to 8 mM, resulted in the formation of a smaller nanoparticle size, while the size increased at 9 and 10 mM concentrations. This effect was attributed to the lack of functional groups available for the reaction when the metal precursor concentration was increased. To achieve the smallest particle size, the middle levels of both metal concentration and temperature were used. Higher temperature resulted in the gradual increase in the NPs size. Unsuitable temperatures lead to increased nanoparticle size and loss of stability, as a result of denaturation, inactivation or low activity of the reductive enzymes and other active molecules involved in the biogenesis process^31,32^. Additionally, it was suggested that the increase in temperature decreases NPs size which is normally due to increment in reaction rate and kinetic energy at higher temperatures^29,33^. Generally, the effect of temperature on the size and stability of the synthesized nanoparticles varies according to the microorganism used^9^. Figure 3B represents the mutual interaction of pH and metal concentration on Alk-ZnO NPs biosynthesis when temperature was fixed at optimum value. The average diameter size decreased gradually with the increment of pH but at higher pH values, the size slightly increased. This may be due to the inactivation of biomolecules involved in the synthesis of Alk-ZnO NPs at low and high pHs^21^. Generally, pH plays a significant role in nanoparticles synthesis, mainly because it has the ability to alter the shape of biomolecules that is responsible in capping and stabilizing the NPs^34^. Also, plays a substantial role in the oxidation state and reducing power of enzymes and secondary metabolites present in cell-free extract^35^. Zhang et al.^36^ stated that pH of the medium solution influences the size and the texture of the synthesized nanoparticles. Modification in pH leads to alteration in the overall charge of bioactive molecules, which in turn facilitates their binding affinity and hence biomineralization of metal ions into nanoparticles^37^. The obtained data reveal that alkaline pH and a high metal concentration are conditions that will generate smallest size of Alk-ZnO NPs. While, a higher or lower pH generated the larger size, which indicated that the catalytic activity of enzymes responsible for Zn^2+^ ions reduction appeared to be deactivated, thus causing an increase in the size of NPs. Figure 3C shows the combined effect of pH and temperature when metal concentration value was fixed at optimum value. It showed that higher values of pH and temperature supported the increase of Alk-ZnO NPs size. The smallest size was obtained in the middle levels of both pH and temperature. The synthesis of small sized ZnO NPs could be attributed to the increase in nucleation process rate as a result of a large number of OH ions. The finding is in agreement with that reported by Mata et al.^38^ who indicated that higher pH favored higher reducing power. Possible reason is that during the elevated pH, the reaction rate will be increased with subsequent crystallization into smaller particles, which involved the nucleation and growth processes of smaller particles from metal nuclei^39^.

The study of physicochemical parameters of the reaction such metal ions concentrations, pH and temperature on Alk-ZnO NPs formation represents the effect of the direct connection between these factors on the particles size. By solving the model regression equation using the SOLVER function of MICROSOFT EXCEL tools, the smallest size Alk-ZnO NPs and the optimum values of these variables can be acquired. The optimal values of the variables were temperature 33 °C, metal concentration 8 mM and pH 8. The model predicts the smallest NPs size that can be obtained using the above optimum conditions to be 20.4 nm.

### Verification of the model

In order to validate the obtained data and to evaluate the accuracy of the applied Box-Behnken statistical design, a verification experiment with optimum concentration of examined variables was carried out in three replicate and compared with the predicted data. The results obtained demonstrated that the average size of nanoparticles was 17.5 nm, while the predicted value from the polynomial model was 20.4 nm. There was a little difference between the experimental and the predicted data. The verification revealed a high degree of accuracy of the model, indicating the model validation under the tested conditions. The measured particle size was less than the predicted by 14.22%. Verification experiment indicated validity, and reliability of response model and applicability of RSM to minimize the size of Alk-ZnO NPs. Additionally, in the present study, *Al.* sp.W7 could synthesize 0.493 g of NPs /L of bacterial supernatant in 24 h.

### Characterization of Alk-ZnO NPs

#### UV–visible spectroscopy

Alk-ZnO NPs synthesized under optimal conditions exhibited the surface plasmon resonance band shifted to a lower wavelength 310 nm, due to their large excitation binding energy^40^. It is known from absorption spectroscopy that as particle size decreases, the band gap energy increases. There is also an opposite ratio between band gap and the absorption wavelength^41^. The corresponding band gap was found to be 4.00 eV. Our data are in good agreement with the earlier reports on biosynthesis of ZnO NPs^25,42^. Aggregation is a common problem with nanoparticles which greatly decreases the surface area and, as a result, affects their physicochemical and biological properties^43^. It is worth to mention that the nanoparticles examined after 10 months using UV–vis spectroscopy showed a peak at 310 nm, indicating their stability (Supplementary Figure S1).

#### Scanning electron microscopy analysis

**Figure 4:**
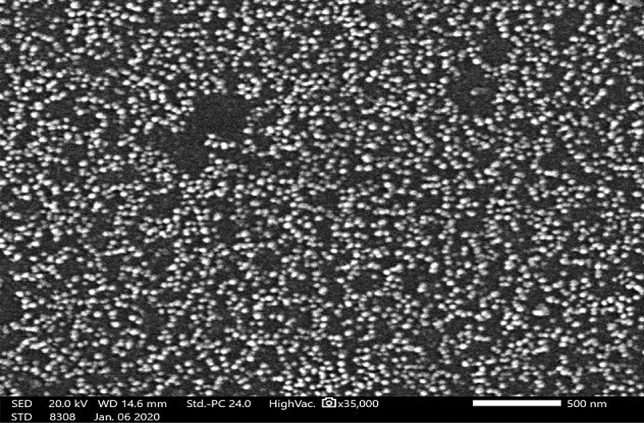
Scanning electron micrograph of Alk-ZnO NPs synthesized under optimum conditions at 35,000 × magnification.

Scanning electron microscopy provided further insight into the morphology and size of the synthesized ZnO NPs. The micrograph illustrated in Fig. 4 proved that they had nano-sized range, smooth, irregular to nearly spherical with a uniform distribution with an average size ranging from 5 to 45 nm.

#### Energy-dispersive X-ray spectrum

**Figure 5:**
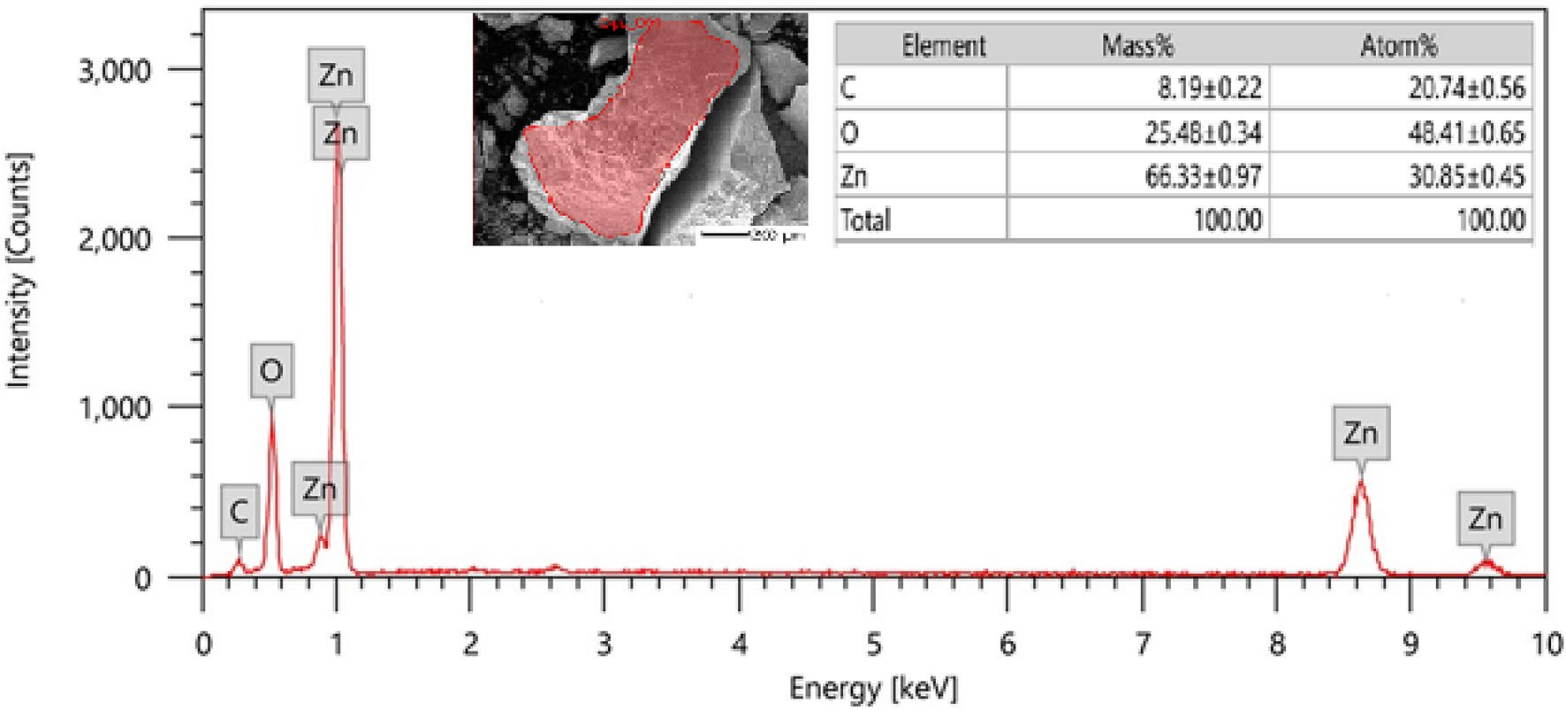
Energy dispersive x-ray spectroscopic analysis of Alk-ZnO NPs. Other elemental signal as carbon was also recorded possibly from enzymes or proteins present in the culture supernatant. Inset image of scanned area and elemental composition.

The Alk-ZnO NPs were subjected to an EDX spectrum to quantify the mixture of metal and oxides present in the sample. Figure 5 clearly illustrates that the EDX spectrum of the fabricated NPs was recorded in the spot-profile mode from one of the densely populated nanoparticles area. Distinct peaks obtained for zinc and oxygen atoms represent the formation of ZnO NPs. The elemental profile showed strong higher peak at 1 keV, characteristic to Zn and confirmed the formation of ZnO NPs^44,45^. The weak signal of C atoms was also recorded and likely due to X-ray emission from carbohydrates/proteins/enzymes present in the bacterial cell free filtrate indicating their involvement in reduction and capping of the synthesized ZnO NPs. The atomic percentages of the elements inset of Fig. 5 reveal zinc as the major element comprising more than 66.33% of total constituent along with oxygen 25.48%, which clearly also confirms the high purity of mediated zinc oxide nanoparticles. Additionally, the optical absorption signals of zinc were observed due to surface plasmon resonance of ZnO NPs^34,45^, which is in agreement with previous reports^46,47^. The impurity free nanoparticles are promising in different application fields as antitumor, anti-microbial and antibiofilm. Our results are in agreement with the results obtained by microbially synthesized ZnO NPs^20,47,48^, where pure form of nanoparticle was clearly depicted through EDX imaging.

#### Transmission electron microscopy

**Figure 6:**
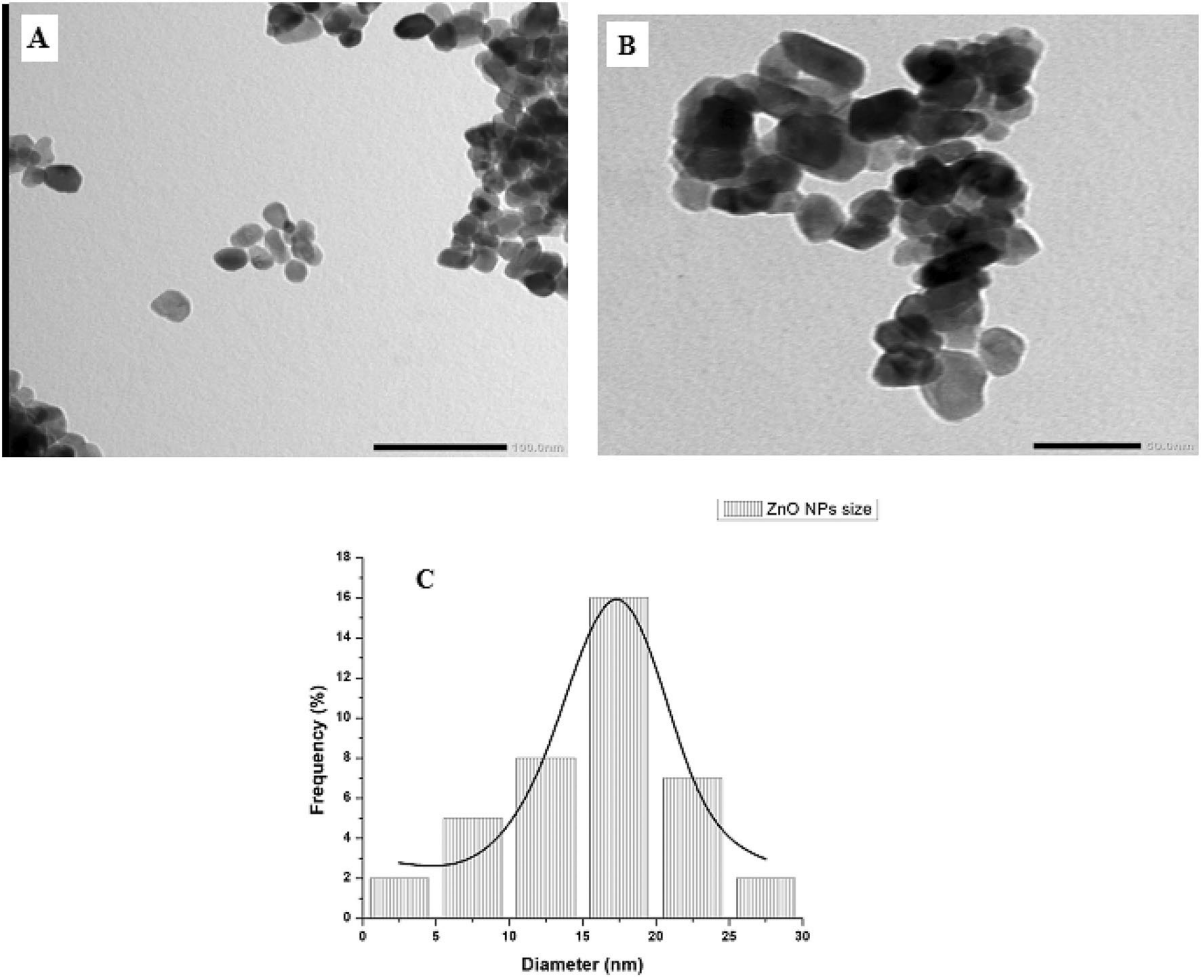
Transmission electron microscopic image of Alk-ZnO NPs (**A**) low magnification (X80k), (**B**) high magnification (X120k), and (**C**) particle size distribution histogram.

Transmission electron microscopic analysis provided exact morphology and size of Alk-ZnO NPs acquired from optimized synthesis conditions and revealed predominantly irregular to nearly hexagonal and quasi-spherical with legitimately agglomeration (Fig. 6A,B). This is a typical phenomenon of interaction of moisture and ZnO and inter-particle interactions (Van der Waals, electrostatic and magnetic forces)^49,50^. The size distribution patterns reveal that the synthesized NPs ranged from 1–30 nm with an average size of 17 ± 1 nm (Fig. 6C). This result is in accordance with crystallite size calculated from the X-ray diffraction. Similar hexagonal—quasi-spherical zinc oxide nanoparticles have been observed in *Rhodococcus pyridinivorans* NT2^46^ and *Lactobacillus plantarum* VITES07^51^. It was previously reported that the smaller the size of ZnO NPs, the greater efficacy in inhibiting the growth of bacteria^52^.

#### X-ray diffraction analysis

**Figure 7:**
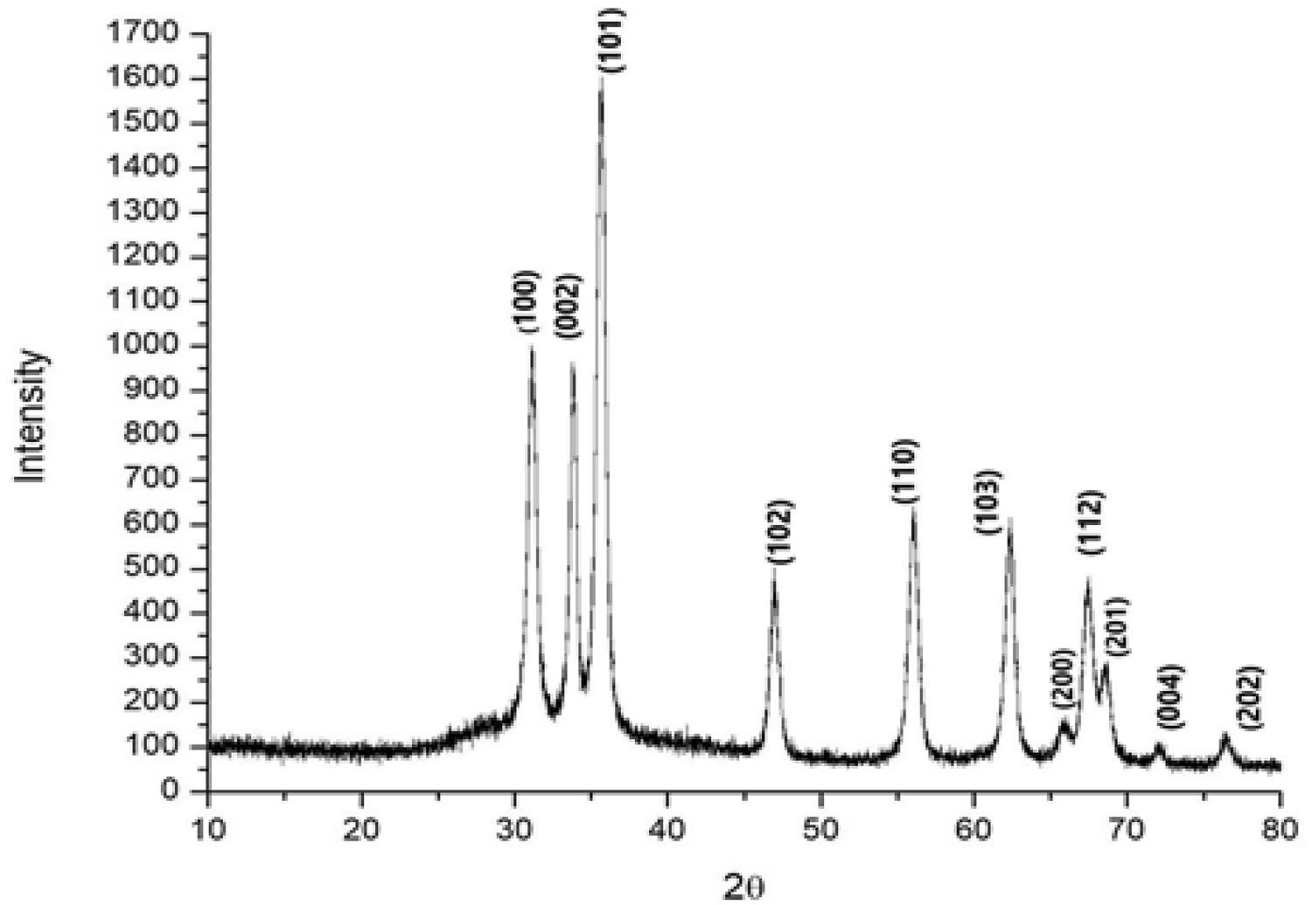
X-ray diffraction pattern of ZnO nanoparticles synthesized by *Al.* sp. W7 supernatant. All peaks indicate purity and crystalline nature. No traces of other impurity phases were detected.

X-ray diffraction is taken in order to further confirm ZnO phase of the nanoparticles. The XRD pattern of the Alk-ZnO NPs fabricated under optimum conditions provided by the RSM having 2θ values with strong diffractions peaks appear at 31.16°(100), 33.83°(002), 35.65°(101), 46.98°(102), 56.05°(110), 62.344°(103), 65.71°(200), 67.46°(112), 68.27°(201), 72.52°(004) and 76.84°(202) (Fig. 7). The XRD diffraction peaks were in good agreement with the reported literature and matched well with wurtzite ZnO of the Joint Committee on Powder Diffraction Standards (JCPDS) Card number 36–1451. Thus XRD pattern shows ZnO NPs with a fine hexagonal crystalline structure formed with significant agreement with this reference file, and no characteristic diffraction peaks were observed other than ZnO, indicating that the bio-assisted NPs were free from other phase impurities and significantly had a high phase purity^13,53^, which was confirmed by EDX analysis results. In addition, the strong and narrow diffraction peaks indicated that the ZnO nanoparticles were of well crystalline structure^46^. Our results are in good agreement with various XRD diffractogram data reported for biologically synthesized ZnO nanoparticles^24,46^. The average crystalline size of NPs corresponding to the most intense diffraction peak at 2θ = 35.65^o^ (101) as calculated by Debye–Scherrer′s equation was found to be 19.5 nm. XRD results confirmed that Alk-ZnO NPs are highly crystalline, having hexagonal wurtzite crystalline structure which are compatible with other researchers’ work^20,55^. XRD results also reflect that the crystallization of the bio-organic phase occurs on the surface of the ZnO NPs. The biosynthesized ZnO nanoparticles shared accord with the crystallite size calculated from TEM.

#### Zeta potential

**Figure 8:**
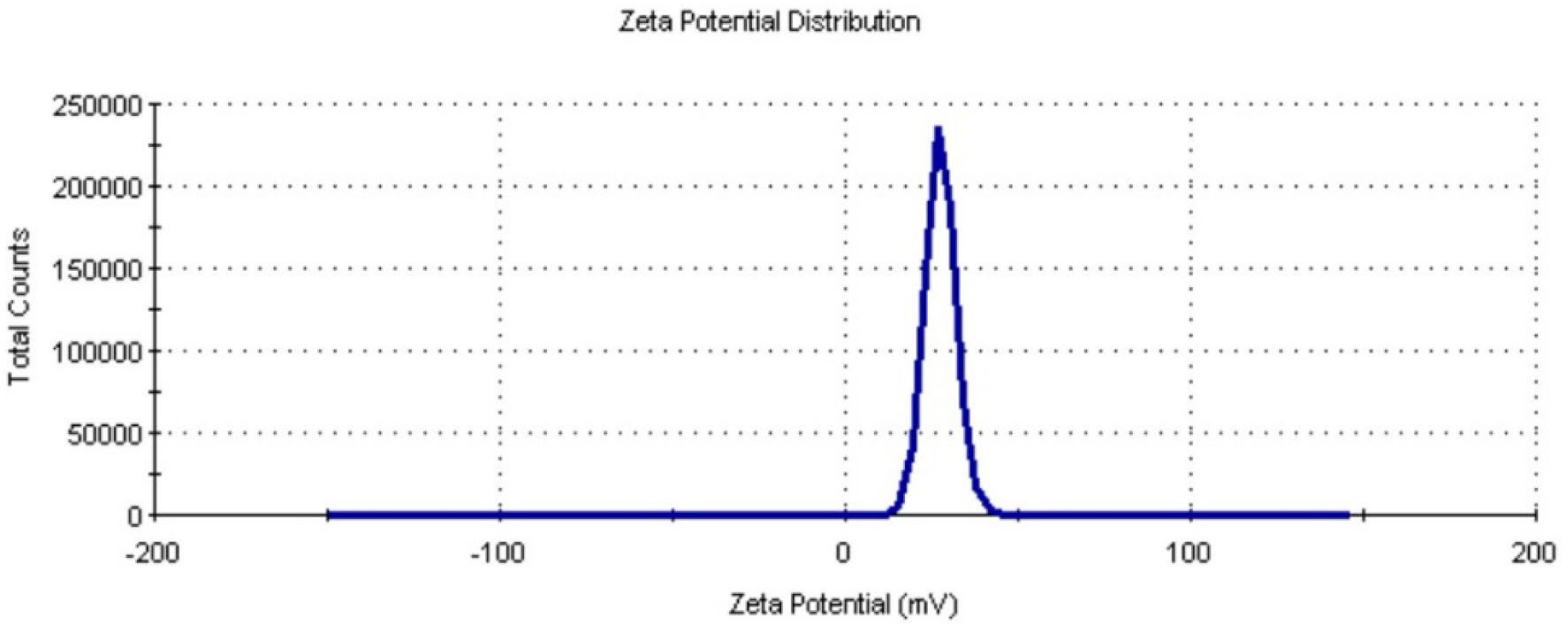
Zeta potential of Alk-ZnO NPs dispersed in water.

Zeta potential of the suspension is one of the key parameters in assessing the stability of the synthesized nanoparticle as it gives the net electrostatic potential of any particle in suspension. The biosynthesized zinc oxide nanoparticles possessed a high positive potential value of 27.5 mv (Fig. 8) which indicates that they were highly stable and prevented agglomeration due to strong electrostatic repulsive forces between them. Nanoparticles with high negative or positive Zeta potential never aggregate due to electrostatic force of repulsion while particles with low zeta potential tend to flocculate^51^. It is generally considered that the zeta potential values (+ 30 mV to − 30 mV) result in good nanoparticle stability^17,51^, this result is comparable with zeta potential of zinc nanoparticles synthesized by *Pseudomonas hibiscicola* (24.64 mV)^24^. Positively charged nanoparticles show better attachment towards negatively charged cell membrane surface due to electrostatic attraction which leads to the penetration of ZnO NPs into the cells and thus enhanced toxicity towards microorganism^56^.

#### Fourier transform infrared

**Figure 9:**
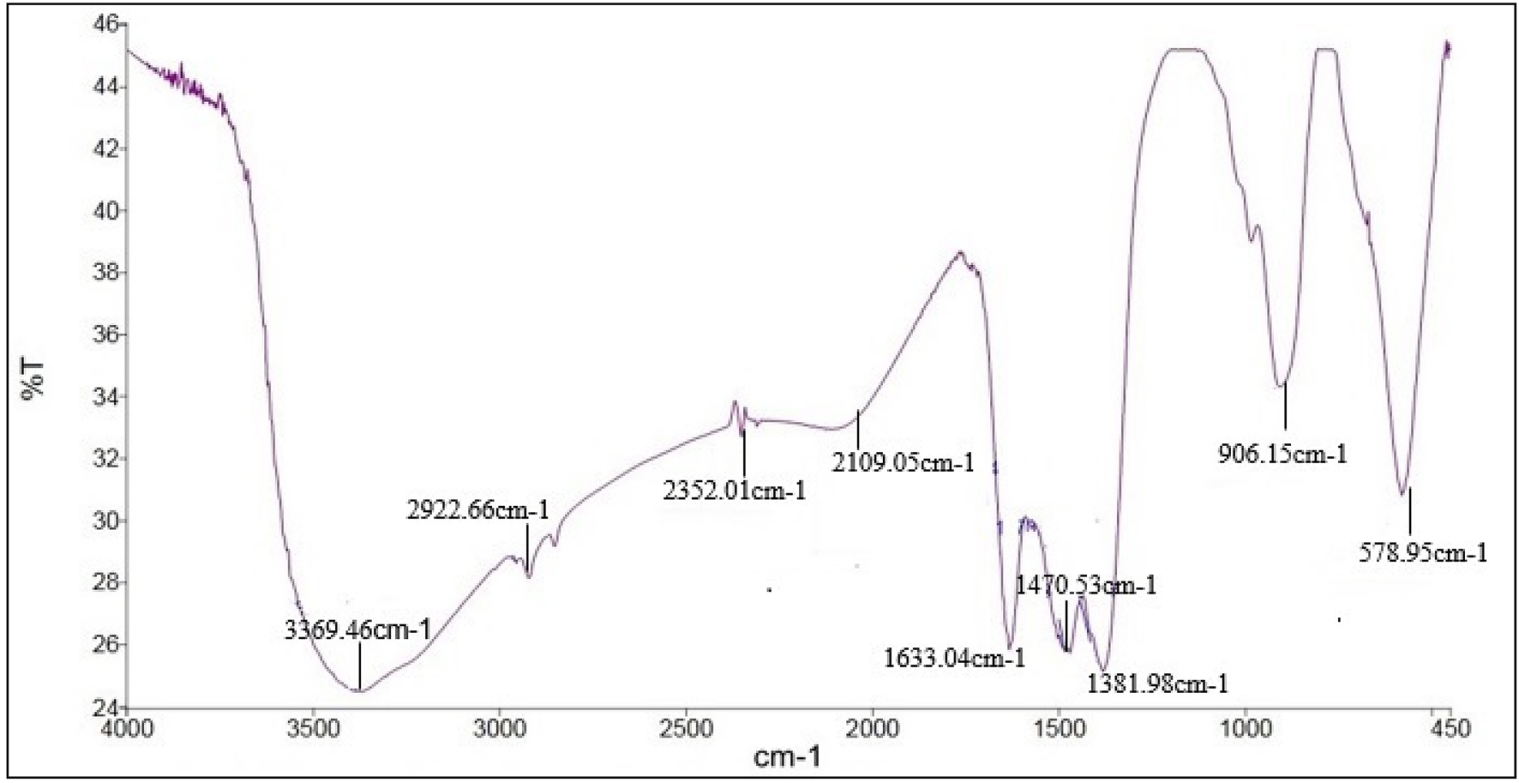
Fourier transforms infrared spectroscopy (FTIR) of Alk-ZnO NPs.

FTIR spectrum was performed to determine the surface chemistry of the ZnO NPs and is used to access the details of functional groups involved in the biomolecules responsible for reducing and capping the bio-reduced ZnO NPs^25,57^. The FTIR spectra for ZnO NPs are presented in Fig. 9. The broad stretching vibrational band with high intensity observed at 3369 cm^–1^ is characteristic for hydroxyl group (OH) or can correspond to the protein N‒H amide I^51^. It is well known that protein-nanoparticle interactions can occur either through free amine groups or cysteine residues in proteins and via the electrostatic attraction of negatively charged carboxylate groups in enzymes^53^. The absorption peak at 2922 cm^−1^ is attributed to C-H stretch of the methylene groups of the protein stretching^21^. Bands at 2352 and 2109 cm^−1^ are associated with the presence of primary amines and sulfur compounds^44^. These peaks indicate the presence of proteins and other organic residues, which might have produced extracellularly by *Al.*sp. W7. Moreover, the absorption peak at 1633 cm^−1^ corresponds to the stretching vibration of N–H bond of primary amines, alkyl C=C aromatic stretching and open chain amino group in proteins^26^. The absorption bands around 1630 cm^−1^ and 1384 cm^−1^ are due to asymmetric and symmetric of stretching carboxylate attached to the ZnO nanoparticles during synthesis^58^. The absorption peaks at 1470 and 1380 cm^−1^ represent the carboxylate group (COO^−^)^59^. According to Tiwari et al.^60^, metal oxides exhibit absorption bands well below 1200 cm^−1^ arising due to interatomic vibrations. Generally metal oxides give absorption peaks in the regions between 500 and 900 cm^−1^^54^. Strong peaks observed at 578.95 cm^−1^ and 906 cm^−1^ prominently indicate Zn–O bond. These findings are in accordance with literature values^20,41^. The stretching mode peaks are indicative of the successful synthesis of ZnO nanoparticles from *Al.*sp. W7 and FTIR spectral analysis reveals the subtle variations in biological components of culture supernatant associated with ZnO NPs formed. The functional groups present in secreted proteins, enzymes and secondary metabolites in strain W7 culture supernatant are responsible for reduction of metal ions and biosynthesis of metallic nanoparticles. The significant reduction capabilities of the biofunctional groups (R-OH, R-NH_2_, R-COO^−^) would reduce Zn^2+^ as electron acceptor to Zn^0^ resulting in the formation of ZnO NPs. These metabolites not only act as reducing agents but also act as capping agents providing stabilization against aggregation. These results are in agreement with previous report in green ZnO NPs synthesis^13,61^.

*Alkalibacillus* sp. W7 nanoparticles showed good antibacterial activity, inhibiting the growth of Gram-negative and Gram-positive bacteria and also efficient photocatalytic degradation of the methylene blue and methyl orange dye under solar irradiation^62^. Moreover, biofilm inhibition activity, anticancer and antioxidant potentials of the synthesized nanoparticles were also investigated.

## Materials and methods

### Microorganism and cultural conditions

*Alkalibacillus* sp. W7 an aerobic Gram-positive, rod-shaped bacterium, isolated from hypersaline Lake Al- Hamra in Wadi An-Natrun, Egypt was used in this study. The 16S rDNA sequence was submitted to NCBI GenBank with an assigned accession number LC164829^63^. The strain was maintained on modified Horikoshi-I medium^64^, containing (g/L): glucose,10; yeast extract,5; peptone,5; K_2_HPO_4_,1; MgSO_4_.7H_2_O,0.2; NaCl, 50; agar, 20; pH 8 and after an incubation period of 48 h at 35 °C was kept at 4 °C and subcultured every month.

### Synthesis of Alk-ZnO NPs

Seed culture was prepared firstly by cultivation of *Alkalibacillus* sp. W7 aerobically in 250 ml Erlenmeyer flask containing 50 mL of modified Horikoshi-1 medium and incubated in a rotatory shaker (130 rpm) at 35 °C for 48 h (OD_600_ = 1.0 ± 0.2). Sterile 50 ml of modified Horikoshi-1 medium in 250 ml Erlenmeyer were inoculated with 2 ml of the fresh inoculum and incubated aerobically on a rotatory shaker incubator for 48 h at 35 °C and 130 rpm. The cell free supernatant (CFS) collected by centrifugation at 5000 rpm for 15 min, then filtered with a 0.22 μm membrane filter (Millipore, USA) was used as a reducing agent for synthesis of Alk-ZnO NPs. Aqueous metal solution of 1 mM ZnSO_4_.7H_2_O in distilled water was used as a precursor. Ten ml of zinc sulphate solution were mixed with 5 ml of *Alkalibacillus* sp. W7 cell free supernatant (2:1, V/V), and incubated for 48 h in dark under static condition at 35 °C to complete the ZnO NPs formation. The color change was observed in the reaction mixture due to the reduction reaction. Simultaneously, control of sterile uninoculated media mixed with precursor salt solution was also maintained at same condition along with the experimental tubes in triplicate.

### Statistical optimization of ZnO nanoparticles biosynthesis

Response surface methodology (RSM) is a collection of mathematical and statistical techniques which has been known as a tool for modeling within minimum number of experimental runs, determining the effects of important parameters and to identify their prominent interactions for the best response^21,23^.

Response surface methodology based on Box-Behnken design^27^ was used to evaluate the relationship between the factors that optimize the response. The combined effect of three active independent variables; pH (X_1_), reaction temperature (X_2_) and Zinc sulphate concentration (X_3_) was considered for evaluation. Their effects were evaluated with respect to responce (NPs size). The levels of each individual variable were selected based on the results of preliminary experiments, involving optimization using the one-factor-at-a-time method. Table 1 shows the experimental Box-Behnken design matrix in the coded and actual levels of selected independent variables developed by Design Expert software (Version 7, Stat-Ease, Inc., USA) and the responce.

### Statistical analysis

Hence, for a three-variable design, a total of 15 experimental runs were performed and their observations were fitted in the second order polynomial model, which is a mathematical expression used to determine the linear, quadratic, and cross effects of the tested independent variables to find the result and approximate the real answer as follows:

$${\text{Y}} =\upbeta _{0} +\upbeta _{1} {\text{x}}_{1} +\upbeta _{2} {\text{x}}_{2} +\upbeta _{3} {\text{x}}_{3} +\upbeta _{12} {\text{x}}_{1} {\text{x}}_{2} +\upbeta _{13} {\text{x}}_{1} {\text{x}}_{3} +\upbeta _{23} {\text{x}}_{2} {\text{x}}_{3}+\upbeta _{11} {\text{x}}_{1}^{2} +\upbeta _{22} {\text{x}}_{2}^{2} +\upbeta _{33} {\text{x}}_{3}^{2}$$

where Y is the dependent variable (size of NPs), X_1_, X_2_, and X_3_ are the independent variables, β0 is the regression coefficient at center point, β_1_, β_2_, and β_3_ are the linear coefficient; β12, β13, and β23 are the second order interaction coefficients, and β11, β22, and β33 are the quadratic coefficients.

### Purification of Alk-ZnO NPs

The nanoparticles were separated from the reaction mixture by high-speed centrifugation at 15,000 rpm for 10 min, then dispersed in sterilized distilled water and centrifuged again. This action was carried out for three times to remove the unreacted metals and the residual biological molecule impurities or any residual metabolites from native nanoparticle samples to obtain purified NPs^6^. Finally, the obtained white color powder was washed with ethanol to remove ionic impurities, then dried overnight in an oven at 40 °C and characterized using different techniques.

### Characterization of biogenic ZnO NPs

#### UV–visible spectroscopy

The biosynthesized ZnO NPs were characterized using UV–Vis spectrophotometer (Thermo Scientific Evolution TM 300) in the wavelength region between 200-nm and 900-nm operated at a resolution of 1 nm and maximum absorbance was determined^65^.

#### X-ray diffraction

X-ray diffraction was performed using Phaser XRD-D2 powder X-ray diffractometer (Bruker, Germany) supplied with Cu-Kα radiation over a wide range of the Bragg angles θ (10° ≤ 2θ ≤ 80°) operated at 30 kV, 10 mA. Data was compared with known standard data published by the Joint Committee on Powder Diffraction Standards (JCPDS Card No. 36–1451). The mean of the crystallite size (nm) was determined from the XRD line broadening measurement using Debye Scherrerr’s equation:

$${\text{D}} = 0.89\uplambda {/}\left( {\upbeta {\text{Cos}}\uptheta } \right)$$

where λ is the wavelength of X-ray radiation, λ = 1.5406 Å, β is the full width at the half maximum (FWHM) of the most intense diffraction peak, and θ is the diffraction angle.

#### Scanning electron microscopy

The shape and size of the ZnO NPs were determined using SEM JEOL IT 200, (JOEL Corp, Tokyo, Japan). The biosynthesized powder was mixed into doubled distilled water and sonicated for 30 min. A small drop of this sample was allowed to dry on a glass slide to make a thin layer of NPs.

#### Energy-dispersive X-ray analysis

The structure of Alk-ZnO NPs was characterized by energy-dispersive analysis X-ray (EDX) spectrum using X-ray micro-analyzer (Module Oxford 6587INCA X-sight) coupled with SEM–IT 200 scanning electron microscope (JOEL Corp, Tokyo, Japan) operated at 20 kV of an accelerating voltage for compositional analysis as well as conformation for the presence of elemental zinc.

#### Transmission electron microscopy

The size and shape of Alk-ZnO NPs were observed using a transmission electron microscope TEM JEM-1400 plus, (JOEL Corp, Tokyo, Japan) operated at an accelerating voltage of 50 kV. A drop of ZnO NPs solution was placed on a carbon coated copper grid followed by water evaporation. The average particles size and the size distribution histogram were determined from microscopic images using a particle size analyzer programe, Image J processing and analysis software (http://imagej.nih.gov/ij/index.html).

#### Zeta potential measurement

The zeta potential was performed by dispersing 1 mg of ZnO NPs in 10 ml of distilled water followed by sonication for 5 min then measurement using Malvern Zetasizer Nano ZS analyzer (Malvern Instruments, Malvern, UK)^66^.

#### Fourier transforms infrared spectroscopy

FTIR analysis was done for identification of presumable biomolecules responsible for the reduction of the Zn^+2^ ions and formation and stability of ZnO NPs. Sample was prepared by mixing Alk-ZnO NPs with potassium bromide KBr using hydraulic press and then dried to remove the moisture content. Infrared spectra were recorded over a range of 450–4000 cm^–1^ with FTIR spectrophotometer (FTIR spectrum Version 10.5.3, Perkin Elmer).

## Conclusions

The present study, built up the first ever account of use of culture supernatant of *Alkalibacillus* sp. W7 towards extracellular synthesis of zinc oxide nanoparticles. The response surface methodology was applied to optimize the conditions influencing the Alk-ZnO NPs size. The results revealed that the optimization of the reaction parameters was essentially needed and directly affected ZnO NPs size. The fitness of the model was verified and predicted values were confirmed. The synthesized Alk-ZnO NPs with optimized conditions showed maximum absorption peak at 310 nm. The XRD results confirmed the efficiency of the synthesis process, evidencing the production of single crystalline NPs with hexagonal wurtzite structure. The average size of synthesized NPs was 17.5 nm, exhibiting quasi-spherical with smooth surface which was confirmed by TEM and SEM analyses. EDX results confirmed the presence of zinc and oxygen with a pure phase. FTIR analysis clearly showed the formation of ZnO NPs and verified the presence of various biomolecules secreted by *Alkalibacillus* sp. W7 acting as capping and stabilizing agents. This green synthesis does not need any additive stabilizer which is a positive point in comparison with chemical methods. Our results confirm the potential of *Alkalibacillus* sp. W7 for ZnO NPs biosynthesis in a simple, fast, cost-effective, convenient and ecofriendly way. Furthermore, the results confirmed that the response surface methodology was beneficial for smaller ZnO NPs size synthesis with great stability over time, and the reaction parameters are directly affecting their size.

## Supplementary Information

Supplementary Information.

## Data Availability

All data produced during this study are included in this published article.
