# Supplementary material for: Statistical optimization of experimental parameters for extracellular synthesis of zinc oxide nanoparticles by a novel haloalaliphilic Alkalibacillus sp.W7
Source: Sci Rep. 2021 May 25;11:10924. doi: 10.1038/s41598-021-90408-y (PMC8149680; doi:10.1038/s41598-021-90408-y)
Supplement: Supplementary file 1 — Supplementary Information. [file 41598_2021_90408_MOESM1_ESM.docx]

(**Supplementary Figure S1**)


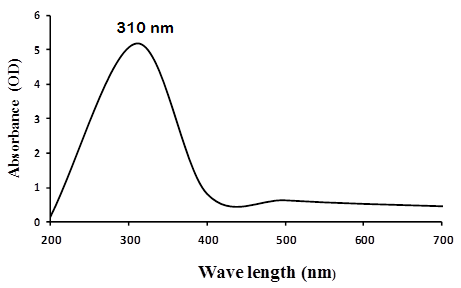


Figure S1. UV–vis absorption spectrum of ZnO NPs under optimal conditions stored for ten months showing a peak at 310.
